# *Lactococcus lactis* Diversity Revealed by Targeted Amplicon Sequencing of *purR* Gene, Metabolic Comparisons and Antimicrobial Properties in an Undefined Mixed Starter Culture Used for Soft-Cheese Manufacture

**DOI:** 10.3390/foods9050622

**Published:** 2020-05-13

**Authors:** Sabrina Saltaji, Olivier Rué, Valérie Sopena, Sophie Sablé, Fatoumata Tambadou, Sandrine Didelot, Romain Chevrot

**Affiliations:** 1 UMR CNRS 7266 LIENSs, Université de La Rochelle, 17000 La Rochelle, France; sabrina.saltaji1@univ-lr.fr (S.S.); vgauthie@univ-lr.fr (V.S.); sophie.sable@univ-lr.fr (S.S.); sandrine.didelot@univ-lr.fr (S.D.); 2 Flores de Terroirs, 17440 Aytré, France; fatoumata.tambadou@flores-de-terroirs.com; 3 INRAE, Université Paris-Saclay, 78350 Jouy-en-Josas, France; olivier.rue@inra.fr; 4 INRAE, BioinfOmics, MIGALE Bioinformatics Facility, Université Paris-Saclay, 78350 Jouy-en-Josas, France

**Keywords:** starter cultures, dairy *Lactococcus*, carbohydrate metabolism, DADA2 algorithm, antimicrobial activities, lactococcin B-like bacteriocin

## Abstract

The undefined mixed starter culture (UMSC) is used in the manufacture of cheeses. Deciphering UMSC microbial diversity is important to optimize industrial processes. The UMSC was studied using culture-dependent and culture-independent based methods. MALDI-TOF MS enabled identification of species primarily from the *Lactococcus* genus. Comparisons of carbohydrate metabolism profiles allowed to discriminate five phenotypes of *Lactococcus* (*n* = 26/1616). The 16S sequences analysis (V1–V3, V3–V4 regions) clustered the UMSC microbial diversity into two *Lactococcus* operational taxonomic units (OTUs). These clustering results were improved with the DADA2 algorithm on the housekeeping *purR* sequences. Five *L. lactis* variants were detected among the UMSC. The whole-genome sequencing of six isolates allowed for the identification of the *lactis* subspecies using Illumina^®^ (*n* = 5) and Pacbio^®^ (*n* = 1) technologies. Kegg analysis confirmed the *L. lactis* species-specific niche adaptations and highlighted a progressive gene pseudogenization. Then, agar spot tests and agar well diffusion assays were used to assess UMSC antimicrobial activities. Of note, isolate supernatants (*n* = 34/1616) were shown to inhibit the growth of *Salmonella* ser. Typhimurium CIP 104115, *Lactobacillus sakei* CIP 104494, *Staphylococcus aureus* DSMZ 13661, *Enterococcus faecalis* CIP103015 and *Listeria innocua* CIP 80.11. Collectively, these results provide insightful information about UMSC *L. lactis* diversity and revealed a potential application as a bio-protective starter culture.

## 1. Introduction

Artisanal soft cheeses are a class of handmade products following ancient practices and manufactured with a limited mechanization process. These cheeses include the use of undefined mixed starter cultures (UMSC) for traditional dairy-based food fermentations [1,2,3]. UMSC are composed of undefined multiple-strain cultures of lactic acid bacteria (LAB). They harbor complex microbiota, which naturally proceed from raw milk, and are specific to the producing region [4]. Most important bacteria in dairy UMSC are *Lactococcus lactis*, *Leuconostoc mesenteroides, Streptococcus* spp. and *Lactobacillus* spp. [1,3,5,6]. However, limited knowledge is currently available on the genetic diversity of UMSC [7]. Changes caused by intrinsic milk properties, but also by other factors such as the dairy environment and cheese making procedures, can influence the UMSC microbial composition. Therefore its complexity is specific beyond the species/subspecies level [8,9,10]. From the beginning of dairying, Lactococci have been widely used as a principal component of starter cultures [6,11]. Both subspecies *L. lactis* subsp. *lactis* and *L. lactis* subsp. *cremoris* are the upmost contributors to the acidification process following the rapid fermentation of lactose [7]. The diversity of *Lactococcus* strains provides an important reservoir for starters and may contribute to flavor differences and specific features in a fermented milk-product [12]. Lactococci participate in food preservation, texture formation and flavor by producing aromatic compounds (alcohols, aldehydes, ketones) or by metabolizing substrates such as citrate, amino acid or fats [13]. The growing interest to preserve traditional cheeses has stimulated the use of raw milk UMSC and thus, a better understanding of the diversity of *Lactococcus lactis* strains is required. High-throughput sequencing (HTS) and whole-genome sequencing (WGS) technologies have opened a new window to decipher such microbial diversity. Lately, industrial and technological relevance has motivated extensive genomic studies [14,15,16] to better predict production performance and advise culture strategies to avoid fermentation failures [17]. Comparative genomics has also provided insightful information into genotype–phenotype associations. Phenotypic data and Lactococci strain-specific information are effective methods for the assignment of functions underlying industrial traits or niche adaptation [18,19]. 

Starter culture use implies the respect of safety and quality patterns in the dairy industry. Spoilage bacteria and foodborne pathogens are of major concern in food production and processing environments as well as for consumers since they cause economic losses and foodborne illnesses. Some LAB species, especially *L. lactis*, can exert a food preservative effect due to a nutritional competition ability or production of organic acids or antimicrobial compounds. The pH reduction makes the dairy matrix unfavorable for the growth of food-spoilage or pathogenic microorganisms. LAB can also produce metabolic end-products including hydrogen peroxide (H_2_O_2_), CO_2_, diacetyl, acetaldehydes, D-isomers of amino acids, reuterin and bacteriocins [20,21]. These antimicrobial strategies are increasingly used for limiting the growth of unwanted microorganisms [22]. In a raw milk starter culture, the food micro-structure is in a liquid form and favors the accumulation of biomass and metabolites causing chemical changes such as a decline of pH. In a gel food microstructure (such as in cheese), microorganisms are immobilized and constrained to form colonies accompanied by local accumulation of metabolic end-products. The immobilization of microorganisms in a cheese sample might result in interactions between colonies and local pH changes to stimulate an acid tolerance response. Therefore, food preservation depends also on the structure of the product [23]. 

In this study, genotype–phenotype associations and bacterial screenings were carried out to characterize the UMSC bacterial diversity to better understand UMSC carbohydrate metabolism and to evaluate antimicrobial activities. 

## 2. Materials and Methods

### 2.1. Sample Collection

The UMSC was obtained following raw milk treatment as described in the Bionatif^®^ patented process [24]. The raw milk was obtained from a farm located in Normandy (Isigny-sur-mer, France), and the UMSC was used daily for soft-cheese manufacture. The UMSC was kept frozen at −80 °C (Flores de terroirs, Aytré, France). 

### 2.2. Gene Sequencing of the UMSC Microbiota

#### 2.2.1. DNA Extraction

Samples of UMSC were aseptically homogenized and centrifuged at 13,000× *g* for 10 min at 4 °C. DNA was extracted with the DNeasy PowerFood Microbial Kit (Qiagen, Hilden, Germany), which combines mechanical (beat beating) and chemical lysis of the cell. The purified DNA was stored at −80 °C. 

#### 2.2.2. Gene Sequencing and Data Analysis of 16S rRNA

The V1–V3 and the V3–V4 regions of the 16S rRNA genes were amplified by PCR. The V1–V3 bacterial universal primers were selected [25,26]. Primers 27F (3′-AGAGTTTGATCATGGCTCAG-5′) and 1492R (3′-TACGGYTACCTTGTTACGACTT-5′) were used for the V3–V4 amplification fragment. For each sample, an amplified DNA quality check was performed. The amplified DNA was then sequenced by the Eurofins Genomics platform (Ebersberg, Germany) using an Illumina^®^ MiSeq Technology. 

The FROGS (find rapidly OTUs with Galaxy solution) pipeline (version 3.1) was used to retrieve the operational taxonomic units (OTUs) under the Galaxy environment [27]. This pipeline includes raw FASTQ files demultiplexing and a preprocess step to filter and delete sequences with unexpected lengths, with ambiguous bases (N) and the ones that do not have primer sequences at both 3′ and 5′ ends. The clustering of sequences was performed by Swarm using an iterative growth process with a local clustering threshold of 3 [28]. Chimera sequences were identified and removed using Vsearch [29]. OTUs accounting for more than 0.5‰ of the total reads were considered. The reference sequences of each OTU were blasted against the SILVA database (version 128) for taxonomic assignation [30].

#### 2.2.3. *purR* Gene Sequencing and Data Analysis

The *purR* gene was amplified by PCR with primers purR-324F (5′-YACTCCATCAAATCTTCGTAAAAT-3′), purR-811R (5′-TGTCATTAAATATATTTCCCAATTGAACA-3′) described by Frantzen et al. [7]. Forward (5′-TCGTCGGCAGCGTCAGATGTGTATAAGAGACAG) and reverse (5′-GTCTCGTGGGCTCGGAGATGTGTATAAGAGACAG) Illumina^®^ adapter overhangs were added to the 5′ ends of primers to enable Nextera XT DNA indexing of the PCR products. The libraries were sequenced using an Illumina^®^ MiSeq technology on the Eurofins Genomics platform (Ebersberg, Germany). The attempted amplicon size was 487 pb [7].

The data were analyzed using the DADA2 pipeline (v. 1.12, http://benjjneb.github.io/dada2/tutorial.html), which retrieves biological sequences from reads by modelling the Illumina-sequencing errors [31]. Once read quality profiles were validated, the core-denoising algorithm of DADA2 was performed on the forward and reverse reads separately. Amplicon sequence variants (ASV) were inferred and pairs merged. ASVs table was constructed and chimeras were identified and removed only if the parent sequence was at least twice abundant than a left/right-segment recombination. The taxonomic affiliation was assigned using a local BLAST database produced using the lactococcal genomes available on the NCBI database. 

### 2.3. Bacterial Library 

Presumptive LAB were enumerated on MRS, BHI or M17 agar (Biokar, Allonne, France) according to the conditions reported in Table 1. Bacterial isolates were isolated (*n* = 1616), purified twice and maintained frozen at −80 °C in the corresponding growth medium containing 20% (*v*/*v*) glycerol (Table 1). 

Each isolate was seeded twice on BHI agar plates (Biokar Diagnostics, Allonne, France), at 28 °C for 30 h.

### 2.4. Phenotypic Profiles and LAB Isolates Identification

#### 2.4.1. Bacterial Carbohydrate Metabolism Profile 

Two isolates issued from growth conditions were randomly selected. Their carbohydrate metabolism profile was determined twice using the API 50 CH system. Overall, it consisted of 49 biochemical tests. API 50 CH was used in conjunction with API 50 CHL medium based on the manufacturer’s instructions (Biomerieux, Marcy l’Etoile, France). 

#### 2.4.2. MALDI-TOF MS Bacterial Identification

MALDI-TOF MS (Biotyper^®^, Bruker, Palaiseau, France) identifications were performed on each bacterial isolate studied for their carbohydrate metabolism profile. MALDI-TOF MS analysis was performed by Eurofins Microbiologie Ouest based on their internal procedures (Nantes, France). 

#### 2.4.3. DNA Extraction, Routine PCR Procedure and Full-Length 16S rRNA Gene Sequencing

Full-length 16S rRNA gene sequencing was performed by the Eurofins Genomics Europe Applied (Ebersberg, Germany) according to the company’s internal test ID BJ00M Bacterial Species Identification. The obtained sequences were aligned using the MEGA7 program package [32] and compared on the NCBI database using the BLAST program (https://blast.ncbi.nlm.nih.gov/Blast.cgi) [33]. 

### 2.5. Genome Sequencing and Data Analysis 

#### 2.5.1. Pure Culture DNA Extraction 

Six *L. lactis* isolates were chosen following API 50 CH results to perform genotype–phenotype associations. Overnight cultures from these isolates were centrifuged at 13,000× *g* for 10 min at 4 °C. These. Genomic DNA was extracted using the DNeasy blood and tissue kit (Qiagen, Germany) according to the manufacturer’s instruction. Genomic DNA was quantified using the NanoDrop ND-1000 (ThermoFisher Scientific, Illkirch-Graffenstaden, France).

#### 2.5.2. Whole Genome Sequencing and Gene Annotation

Whole-genome sequence data were generated by PacBio^®^ RS II and Illumina^®^ iSeq sequencing technology in order to produce de novo assemblies. The PacBio^®^ RS II data were obtained and assembled using the Hierarchical Genome Assembly Process (HGAP) assembly algorithm [34], on the Eurofins Genomics platform (Ebersberg, Germany). The Illumina^®^ iSeq data were produced and assembled using the SPAdes algorithm (Mérieux Nutrisciences, Nantes, France) [35]. Chromosomal organization of the resulting contigs were predicted by aligning them against the *L. lactis* subsp. *lactis* IL1403 reference genome using the MicroScope platform [36]. The individual genome assemblies were deposited in the NCBI database with accession codes listed in Table 2. Overall genomic relatedness between genomes was assessed using the Orthologous Average Nucleotide Identity (OrthoANI) tool [37]. Carbohydrate metabolism-related pathway information was identified from those described in the Kyoto Encyclopedia of Genes and Genomes (KEGG, http://www.kegg.jp/) [38]. The mining of genes clusters related to the production of antimicrobial peptides was performed using BAGEL4 [39]. 

### 2.6. Accession Number(s)

The 16S and *purR* amplicon data were deposited at DDBJ/ENA/GenBank under BioProject number PRJNA615620. The whole-genome project was deposited at DDBJ/ENA/GenBank under BioProject number PRJNA615395.

### 2.7. Antimicrobial Activity

#### 2.7.1. Bacterial Strains and Growth Conditions

Indicator strains were selected from culture collections and maintained at −80 °C in their growth medium supplemented with 20% glycerol (*v*/*v*). They were cultured twice in growth conditions specific to each strain (Table 3). 

#### 2.7.2. Determination of Inhibitory Properties of LAB Isolates against Foodborne Pathogenic Bacteria and Spoilage Bacteria

##### Agar Spot Test

An agar spot test was used to determine the antimicrobial potential of LAB isolates against the indicator strains (Table 3). Five microliters of an overnight culture (approximately 10^8^ CFU/mL) of each LAB isolate were spotted onto BHI agar plates (15 g/L agar) and left to dry for 20 min at room temperature under sterile conditions. The plates were then overlaid with 10 mL of soft agar (8 g/L) seeded with 10^6^ CFU/mL of the indicator strain (Table 3). Plates were incubated overnight at the optimum growth temperature of each indicator strain and checked for inhibition halos. As positive control, *Lactobacillus sakei* CIP 104,494 was used because of its large antimicrobial spectrum [41]. 

##### Preparations of Cell-Free Supernatant (CFS) and Neutralized CFS (NCFS)

Cell-free supernatants (CFSs) were obtained by centrifugation at 8600 g for 10 min at 4 °C to separate bacterial cells and supernatants. The neutralized CFS (NCFS) were prepared by adjusting the pH of CFS to 7.0 with NaOH 1M to exclude the antimicrobial effect of organic acids. Samples were heated at 100 °C for 10 min to inhibit the enzyme activity. The antimicrobial properties of CFSs and NCFSs were tested on the indicator strains by the agar well diffusion assay.

##### Agar Well Diffusion Assay

BHI agar was seeded with 10^6^ CFU/mL of indicator strains (Table 3), homogenized and poured into sterile Petri dishes. After drying under sterile conditions, sterile glass wells were placed on agar plates. Aliquots of non-diluted CFS or NCFS produced by LAB isolates were used to fill in the wells (100 µL). Plates were incubated overnight at the optimum growth temperature of each indicator strain. Inhibition halos were observed and recorded. 

##### Inhibitory Effect during Co-Cultivation

The ability of LAB isolates to inhibit the growth of *S. aureus* DSMZ 13,661 and *S.* ser. Typhimurium CIP 104,115 was further studied during co-cultivation assays in a double concentrated BHI media (pH 7) [42]. The co-cultivation process consisted of two different culture batches. Batch 1 corresponded to the co-inoculation of *S. aureus* DSMZ 13,661 with the selected LAB isolate and batch 2, the co-inoculation of *S.* ser. Typhimurium CIP 104,115 with the LAB isolate. Growth control was performed using a single culture for each indicator strain or the LAB isolate. An inoculum of 1% (*v*/*v*) of LAB isolate overnight culture (10^9^ CFU/mL in BHI broth) and 1% (*v*/*v*) of indicator strain overnight culture (10^9^ CFU/mL in BHI broth) were seeded to perform Batch 1 or Batch 2 co-culture assay. All batches were incubated at 28 °C for 48 h. The CFS from each batch culture was collected every 2 h. Antimicrobial activities were detected by the agar well diffusion assay as described above. 

## 3. Results and Discussion 

### 3.1. UMSC Bacterial Diversity Studies 

Cheese manufacture behavior, processing environment and starter cultures are known to harbor an indigenous microbiota that influences organoleptic features on artisanal cheeses. The bacterial composition of starter cultures supports organoleptic and biochemical changes during ripening [43]. Revealing information on microbial consortia are essential for artisanal cheesemakers, considering the willingness of the consumer market to require locally manufactured products with different organoleptic properties [9,43,44]. Part of a daily routine, the studied UMSC is used for soft-cheese manufacture. Its microbial transfer is applied during rennet addition, a few hours after milk enrichment with acidifying starters. This cheese-making practice, combined with a high diversity of microbial activities, is key to developing gustatory characteristics. In this study, the microbial consortium of the UMSC was deciphered using culture-independent and culture-dependent based methods. 

First, the UMSC microbial diversity was assessed using three different culture-independent methods: (i) analysis of 16S rRNA gene fragment sequences, (ii) *purR* gene sequence analysis, (iii) whole-genome sequencing analysis. Afterward, two culture-based methods were used on bacterial isolates recovered from the UMSC: MALDI-TOF MS and full-length 16S rRNA gene sequencing analysis. 

#### 3.1.1. Analysis of 16S rRNA Gene Fragment Sequences

An assessment of the microbial diversity started with targeted amplicon sequencing of 16S rRNA gene fragments. Read counts (total of 65,447 reads for V3–V4 and 61,383 reads for V1–V3) were filtered and relative abundances were calculated for each OTU. One dominant genus, *Lactococcus* sp., encompassing 99.9% of relative abundance was identified for each 16S rRNA gene region. With a clustering threshold of three, two OTUs were obtained among the *Lactococcus* genus. 

#### 3.1.2. *purR* Gene Sequence Analysis

The housekeeping gene *purR* was then studied to complete the assessment of microbial diversity. It was previously selected after pan-genome analysis for the differentiation of clades beyond the *L. lactis* subspecies level [7]. The *purR* gene encodes for a transcriptional regulator of purine biosynthesis. The use of a single nucleotide inference method highlighted five amplicon sequence variants (ASVs) after raw data processing using the DADA2 algorithm (Table 4). A total of 108,455 reads were obtained with an exact *purR* amplicon size of 435 nucleotides for each clustered ASV [45]. 

**Figure 1:**
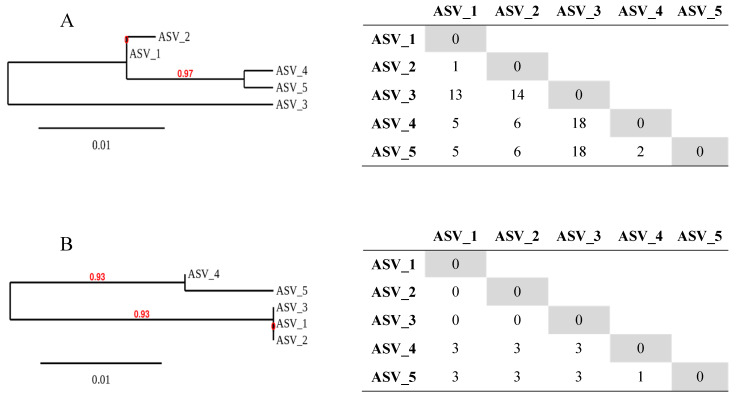
Dendrograms showing sequence similarities of *L. lactis* strains based on a cluster analysis of the *purR* amplicon nucleotide variants (**A**) or amino acid variants (**B**). *purR* genes were sequenced from the undefined mixed starter culture (UMSC) global genomic DNA extraction. The tree was constructed using Phylogeny.fr [46]. Matrix tables highlight differences in the composition of nucleotide (**A**) or amino acid (**B**) sequences.

A close strain similarity was revealed and each ASV variant was represented by a single nucleotide sequence type. Nucleotide variant analysis showed that the most abundant sequence (ASV_1) was 100% identical to the corresponding reference sequence *L. lactis* subsp. *lactis* UC08 (GenBank: CP015903.1) or UC11 (GenBank: CP015904.1). A matrix table highlighted nucleotide substitutions between each ASVs, from 1 up to 18 different bases (Figure 1A). The nucleotide translation into amino acid sequences identified three different ASVs (see matrix table, Figure 1B). Amino acids sequences of variants ASV_1, ASV_2 and ASV_3 were identical. Variants ASV_4 and ASV_5 showed three amino acid substitutions compared to other ASV (or the reference sequence). A comparison between ASV_4 and ASV_5 showed one amino acid substitution. Substitutions include amino acid changes in position 7: Glu (ASV_1), Ala (ASV_4) and Ser (ASV_5), position 21: Thr (ASV_1) and Pro (ASV_4 and ASV_5) and position 22: Lys (ASV_1) and Gln (ASV_4 and ASV_5). Dendrograms were constructed using nucleotide alignments (Figure 1A) or protein alignments (Figure 1B). 

These five ASVs sequence reads were all affiliated to the species level (100% identity) as *L. lactis* using BLAST searches against a local database including any sequences of lactococcal genomes part of the NCBI database (built on 10 February 2020). Interestingly, the phylogenetic analysis of *purR* did not identify *L. lactis* to the subspecies level, as it did for Frantzen et al. [7]. In their work, primers designed by Frantzen et al. were used for relative quantification of the microbial community in starter cultures. Medium amplicon size was then required. Therefore, the *purR* amplicon size (435 nucleotides) was shorter than the full-length *purR* gene (816 nucleotides), identified in the whole-genome sequences. The targeted amplicon *purR* showed an alignment between position 347 to 782. A BLAST search performed on the full-length *purR* gene affiliated the genome sequences to the subspecies level (100% identity) as *L. lactis* subsp. *lactis*. These results suggested that new primers devised to amplify longer fragments of the *purR* gene would be needed to explore *L. lactis* diversity to the subspecies level in the UMSC. Another approach was attempted using the *eps* operon, both located on plasmids and chromosomes [7]. Unfortunately, irregular amplicon sizes were obtained, and it was impossible to differentiate isolates to the species/subspecies level (data not shown).

The ASV method infers the biological sequences in the sample before the introduction of amplification and sequencing errors. It distinguishes sequence variants differing by as little as one nucleotide [47]. The OTU assignment methods gather clusters of reads that differ by less than a fixed clustering threshold of three [28]. In a previous study, Lactococci strains were confirmed to have multiple copies of the 16S rRNA gene. These copies harbor intra-genomic heterogeneity, which makes it difficult to differentiate bacteria to the species/subspecies level using 16S rRNA amplicon [45,48]. ASV methods better discriminate ecological patterns capturing all biological variation [31]. Furthermore, DNA sequences of protein-coding genes are more effective than 16S rRNA genes when distinguishing between very closely related bacteria [7,49]. The targeted amplicon comparison highlighted a noteworthy difference between results obtained with fragments of the 16S rRNA gene (2 OTUs) or with the *purR* gene (5 ASVs). The housekeeping *purR* gene seems to be the best candidate since it enabled the differentiation of clades beyond the species level. 

#### 3.1.3. Whole-Genome Sequencing Analysis

The ANI value is used to estimate the genetic distance between isolates, which reflects the sequence identities of the conserved regions between two genomes [50]. Whole-genome sequencing and assemblies were performed. The pairwise OrthoANI values of six in-house *Lactococcus* and one reference genome (*L. Lactis* subsp. *lactis* IL1403) were calculated to assess their genetic relatedness. One cluster was retrieved (Supplementary Materials Figure S2). Based on the guidelines set out by Richter and Rosselló-Móra, *L. lactis* subsp. *lactis* strains are a different species to subspecies *cremoris* strains possessing an ANI value below 95% [51]. Here, the ANI identity was >99.8% for all of the *L. lactis* genomes, suggesting they all belong to the *lactis* subspecies group. 

#### 3.1.4. Bacterial Library 

Second, a culture-dependent based method was performed to complete the study of the UMSC bacterial diversity. A bacterial library was established from the UMSC with a set of 1616 bacterial isolates, according to 13 growth conditions (Table 5). 

#### 3.1.5. MALDI-TOF MS and Full-Length 16S rRNA Gene Sequencing Analysis

Two representative isolates of each growth condition were randomly selected for MALDI-TOF MS and full-length 16S rRNA gene sequencing characterizations. A set of 26 bacterial isolates (*n* = 26/1616) was analyzed as a sample that could represent the bacterial diversity of each growth condition. Four species identifications were obtained: *Lactococcus lactis* (L) (*n* = 18/26), *Lactococcus raffinolactis* (R) (*n* = 1/26), *Enterococcus faecalis* (E) (*n* = 5/26) and *Staphylococcus warneri* (S) (*n* = 2/26). The culture-dependent analysis showed similarities in the UMSC diversity and agreed with culture-independent studies for the *L. lactis* identification. Results also highlighted the presence of other species besides *L. lactis*. These latter could be part of the remaining 0.1% of relative abundance outlined by 16S rRNA targeted amplicon sequencing. In terms of genus richness and effective number of genera, the differences observed among 16S rRNA analytical method were most likely a result of unclassified reads in the bioinformatic pipeline [52]. 

### 3.2. UMSC Strains Metabolic and Functional Characteristics

#### 3.2.1. UMSC Genotype–Phenotype Associations

API 50 CH systems were used to explore carbohydrates metabolism. Height carbohydrate metabolism profiles were retrieved from the previous 26 examined LAB isolates (Supplementary Materials Figure S1). Three dairy *L. lactis* type strains were also tested (Table 6). Carbohydrate metabolism plays a key role in the growth of *Lactococcus* species. On average, 12.5% of genes are associated with carbohydrate metabolism and involved in glycolysis/gluconeogenesis and the pentose phosphate pathway (PPP) [53]. Four *L. lactis* phenotypic groups were obtained: (A) for isolates L1 to L6, L10 to L13 and L15 to L17 (*n* = 13); (B) for isolates L7 and L8 (*n* = 2); (C) for isolates L14 and L18 (*n* = 2) and (D) for isolate L9 (*n* = 1) (Table 6).

A genotype–phenotype association was devised to study possible relations between genes and phenotypes observed in the API 50 CH systems. A representative set of six *L. lactis* isolates were selected for WGS studies: three isolates were part of the A group (L1, L2, L3), one isolate was part of the B group (L8), one isolate was part of the C group (L14) and one isolate was part of the D group (L9). An Illumina iSeq^®^ platform (L1, L2, L8, L9, L14 genomes) and a PacBio^®^ RS II technology (L3 genome) were used for genome assemblies. The role of *L. lactis* as an important industrial starter strain is mainly due to the rapid conversion of lactose to lactic acid. The KEGG pathways (https://www.genome.jp/kegg-bin) of Lactococci cell metabolism summarize two possible catabolic ways for lactose utilization: (i) hydrolysis of lactose to give α-D-glucose and D-galactose performed by a β-galactosidase (EC 3.2.1.23), and (ii) the conversion into a lactose 6-phosphate (Lac-6P) by a lactose phosphotransferase (EC 2.7.1.207). The Lac-6P is subsequently hydrolyzed by 6-phospho-β-galactosidase (EC 3.2.1.85) to give D-galactose-6P (Gal-6P), and then Gal-6P is catabolized by the tagatose 6-phosphate pathway [55]. Genome sequence analysis highlighted both catabolic pathways with genes encoding these key enzymes. Conversely, phenotypic results indicated that the B group was able to degrade D-sorbitol, although it lacked all gene families found to be significantly associated with this degradation. Buron-Moles et al. reported the same results with dairy *L. lactis* isolates [14]. 

Regarding D-tagatose degradation, phenotypic results observed in the API 50 CH systems revealed that isolates of the B group could use this carbohydrate source whilst other isolates (or type strains) could not. D-tagatose is hydrolyzed by a tagatose-6-phosphate kinase (EC 2.7.1.144) to give D-tagatose-6P (Tag-6P). Tag-6P is subsequently hydrolyzed by a tagatose-1,6-diphosphate aldolase (EC 4.1.2.40) to give Tag-6P_2_, and then it enters in the PPP. These enzymes were found in each studied genome even though the B group was the only one able to grow on D-tagatose. By studying genotype–phenotype associations between gene families and their carbohydrate metabolism, Buron-Moles et al. reported that D-tagatose can only be degraded by a few LAB members of *Lactobacillus* and *Pediococcus* strains [14]. Siezen et al. also reported the inability of *Lactococcus* strains to grow on D-tagatose [54]. Here, the *L. lactis* isolates were highlighted for their uncommon D-tagatose degradation. 

D-xylose units are components of the β-1,4-xylan, a hemicellulosic polysaccharide of plant materials [54]. The β-1,4-xylosidase (EC 3.2.1.37) is a key enzyme involved in the β-1,4-xylan degradation to obtain D-xylose oligomers. D-xylose is then converted by a xylose isomerase (EC 5.3.1.5) to form D-xylulose (D-xyl). D-xyl is then catabolized by a xylulokinase (EC 2.7.1.17) to enter in the PPP. Genes encoding these enzymes were found in each analyzed genome. The carbohydrate metabolism study demonstrated that isolates were able to use D-xylose, except for those that were part of the B group, the *cremoris* subspecies type strain and the dairy strain *L. lactis* IL1403. Similarly, phenotypic results highlighted isolates able to grow on D-sucrose except for group A and *L. lactis* type strains, which were sucrose-negative. A previous study also reported the inability of dairy *Lactococcus* strains to utilize D-sucrose [14], unlike strains from plant origin that could grow on the D-sucrose [18,54]. Genotypic relationships showed no genes encoding for D-sucrose degradation. As noted by Buron- Moles et al., these findings emphasize that phenotypes cannot be always inferred from the presence/absence of specific genes but require the explicit integration of phenotypic information [14]. 

Phenotypic features in carbohydrate metabolism appeared to reflect the functional diversity observed for each *L. lactis* isolate. Moreover, the percentage of carbohydrates utilized per isolate was slightly higher (33% to 45%) in comparison with *L. lactis* type strains (29%). Their metabolism potential exhibited differences and seemed to be richer and diversified. Interestingly, the presence of some genes did not always imply a degradation of the associated carbohydrate in the API 50 CH systems. It could be explained by a progressive pseudogenization process (gene inactivation) linked to their current niche specification. The *L. lactis* isolates came from a farm environment before being introduced into raw milk and then into the UMSC. A comparative study revealed that an *L. lactis* gene set related to degradation products such as arabinose and xylose originated from plants [54]. Genomes of subspecies *lactis* KF147 and KF282, which were of plant origin, possessed genes set for the uptake and conversion of typical cell wall degradation products such as arabinose and xylose. All of the analyzed genomes possessed a key enzyme for L-arabinose degradation even though phenotypic profiles showed an inability to grow on arabinose (Table 6). Moreover, B group isolates possessed the *araD* gene, encoding an L-ribulose-5-phosphate 4-epimerase (EC 5.1.3.4), but it was annotated as a pseudogene. A mutation may have divided the gene into two-parts, and thus the *araD* gene became inactive. These results suggested that the human-made environment triggered microbial strains to genomic function adaptations to their dairy environment. UMSC isolates progressively appeared to be domesticated since they no longer could grow in a variety of plant-derived carbohydrates due to pseudogenization or transposon insertions in genes [56]. These genomic changes could be worsened due to the “backslopping” process in which artisans are continuously transferring microbial material from the previous fermentation product to start a new batch [15]. 

#### 3.2.2. Antimicrobial Activities

The antimicrobial activity of bacterial isolates against foodborne, food-spoilage and pathogenic bacteria was evaluated by the agar well diffusion assay. All isolates that were part of the bacterial library (*n* = 1616) were tested. A total of 34/1616 isolates showed antimicrobial activity against up to five indicator strains (Table 7 and Supplementary Materials Table S1). Isolates 14 and 15 showed the largest antimicrobial spectrum inhibiting the growth of the five indicator strains (*S.* ser. Typhimurium CIP 104115, *S. aureus* DSMZ 13661, *S. aureus* CIP 76.25, *E. faecalis* CIP 103,015 and *L. innocua* CIP 80.11). *S. aureus* DSMZ 13,661 was sensitive and showed the largest inhibition zones. *S.* ser. Typhimurium CIP 104,115 was the only Gram-negative bacteria to be sensitive toward isolates 14 and 15. The antimicrobial activity of the *L. lactis*-isolate 14 was further studied in co-cultivation assays against the two indicator strains: *S. aureus* DSMZ 13,661 or *S.* ser. Typhimurium CIP 104115. 

During co-culture assays, the growth of each bacterium was monitored by bacterial counts on a specific medium (data not shown). Growth controls were obtained using bacterial counts performed on single cultures of each strain (indicators and isolate 14). Temporary growth slowdowns were observed during co-cultures and compared with their respective growth controls. Growth slowdowns in co-culture assays were highlighted in the exponential growth phase of *S.* ser. Typhimurium (between 8 and 20 h) and in the stationary growth phase of *S. aureus* DSMZ 13,661 (between 26 and 34 h). Their respective controls showed no growth disruption. The growth in the co-culture of isolate 14 remained similar to that of the growth control containing no indicator strain (data not shown). To further examine isolate 14 antimicrobial activity, CFSs of each batch culture were collected during these growth slowdowns. Their antimicrobial activity was assessed by the agar well diffusion assay. Thus, CFSs were collected between 26 and 34 h of growth for *S. aureus* DSMZ 13,661 assays (mono and co-cultures). Their CFSs largest inhibition halo was obtained after 32 h of growth. Similarly, CFSs were collected between 8 and 20 h of growth for *S.* ser. Typhimurium CIP 104,115 assays (mono and co-cultures). Their CFSs largest inhibition halo was obtained after 16 h of growth (data not shown). Table 8 summarizes inhibition halo values (mm) of CFSs both obtained from mono and co-cultures after 32 h of growth for *S. aureus* assays and after 16 h of growth for *S.* ser. Typhimurium assays. 

CFSs of isolate 14, alone or in the presence of indicator strains, showed antimicrobial activity against *S*. ser. Typhimurium CIP 104115, *Lactobacillus sakei* CIP 104494, *S. aureus* DSMZ 13661, *L. innocua* CIP 80.11 and *L. lactis* subsp. *lactis* IL1403. CFSs of indicator strains showed no inhibition halo against themselves. These results highlighted an increase in inhibition halo sizes when isolate 14 was co-cultivated with *S. aureus* or *S*. ser. Typhimurium. The recorded pH values between 8 and 20 h and 26 and 34 h of incubation time ranging from 6.6 to 6.8 for each batch culture. Comparative studies described the growth of *S. aureus* under much more stringent conditions, at pH values of 5.25 and 4.48 [58] or 5.6 in milk [59]. The inhibition of *S. aureus* in buffered media by *Lactococcus* strains was also observed at pH 6.8 and cannot, therefore, be attributed to a pH drop since *S. aureus* can grow at lower pH values [59]. Mufandaedza et al. also reported the survival and the growth of *Salmonella* sp. in fermented product associated with low pH [60]. 

The web-based tool BAGEL4 was used to mine secondary metabolite gene clusters within the whole genome *L. lactis* L3 (isolate 14). A coding sequence sharing 51.35% identity with the *lcnB* gene was recovered. The gene is known to be involved in the production of the lactococcin B bacteriocin (LcnB) [61]. A sequence sharing 69.23% identity with the *lciB* gene was also recovered and is involved in the immunity toward LcnB. A putative export system encoded by analogs of *lcnC* and *lcnD* genes was also identified with 98.37% identities. This analysis highlighted the presence of four genes sharing identities with the lactococcin operon [62]. The LcnB affects membrane permeability, dissipating its potential and its pH gradient caused by leakage of ions. In *L. lactis* strains carrying the immunity protein, the LcnB is inactive [63]. The LcnB antimicrobial activity was detected against close-related Lactococci strains. None of the Lactococci strains LcnB producers could inhibit the growth of other Gram-positive or Gram-negative bacteria [64,65]. Isolate 14 inhibited the growth of *L. lactis* IL1403, a plasmid-free strain sensitive to bacteriocin. These results suggested that isolate 14 could produce an antimicrobial peptide close to the LcnB. Şahİngİl et al. also reported inhibition of *Salmonella* sp. by a lactococcin-like bacteriocin, produced by an *L. lactis* subsp. *lactis* strain [66]. That lactococcin activity was observed during the early logarithmic phase (6–7 h of growth) and had maximal activity at 18 h of incubation time, as with the results shown here for isolate 14. 

## 4. Conclusions

This study described the microbial community of an undefined starter culture with its richness in *Lactococcus* strains, naturally present in the raw milk microbiota. Five *L. lactis* variants were identified, and five carbohydrate metabolism profiles were highlighted in the *Lactococcus* genus. These *Lactococcus* strains differ in their phenotypic properties from the strains commonly used as industrial starters. Moreover, some *L. lactis* isolates presented an antimicrobial activity detected by cultural methods. These bacteria could act as a biocontrol agent when combined with other procedures in the so-called hurdle technology. Their GRAS status, their origin from an ecological niche and their role in flavor formation support the use of the UMSC in cheese manufacture procedures. Furthermore, considering the culture characteristics and their relationship with product quality, further studies could be helpful to determine their effects on technological aptitude and sensory properties of the cheese. 

## Figures and Tables

**Table 1:** Growth conditions used for lactic acid bacteria (LAB) and non-LAB isolation.

Growth Conditions		
Aerobic atmosphere	28 °C	BHI agar
		MRS agar
		M17 agar
		MRS agar + 2% NaCl
		MRS agar + 4% NaCl
		MRS agar + 6.5% NaCl
		BHI agar + 6.5% NaCl
Microaerophilic atmosphere	28 °C	BHI agar
		MRS agar
		M17 agar
Aerobic atmosphere	22 °C	BHI agar
	37 °C	MRS agar
	44 °C	MRS agar

**Table 2:** Genomic features of subspecies in the genus *Lactococcus*.

Strains	Sequencing Technology	Scaffold Number	Genome Size (Mb)	GC %	Predicated Coding Genes	GenBank Accession Number
*L. lactis* subsp. *lactis* L1	Illumina^®^ iSeq	177	2.75	34.96	2945	JAATTT000000000
*L. lactis* subsp. *lactis* L2	Illumina^®^ iSeq	193	2.78	34.99	3003	JAATTS000000000
*L. lactis* subsp. *lactis* L3	PacBio^®^ RS II	7	2.84	35.22	3024	JAATTU000000000
*L. lactis* subsp. *lactis* L8	Illumina^®^ iSeq	242	2.81	34.99	3036	JAATTR000000000
*L. lactis* subsp. *lactis* L9	Illumina^®^ iSeq	229	2.80	34.98	3014	JAATTQ000000000
*L. lactis* subsp. *lactis* L14	Illumina^®^ iSeq	178	2.78	34.98	2966	JAATTP000000000
*L. lactis* subsp. *lactis* IL1403	[40]	[40]	2.37	35.33	2473	AE005176.1

**Table 3:** Growth media and growth conditions of food-spoilage and foodborne bacteria used as indicator strains.

Indicator Strains	Strain	Culture Collection	Growth Medium	Growth Conditions (°C)	Microaerophilic Condition
*Bacillus cereus*	CIP 66.24T	CIP ^1^	BHI ^5^	30	No
*Bacillus subtilis*	CIP 52.65T	CIP	BHI	30	No
*Brochothrix thermosphacta*	DSMZ 20171T	DSMZ ^2^	BHI	25	No
*Carnobacterium maltaromaticum*	CIP 103158	CIP	MRS ^6^	30	Yes
*Cronobacter sakazakii*	CIP 103183T	CIP	BHI	30	No
*Escherichia coli*	DSMZ 1103	DSMZ	BHI	37	No
*Enterococcus faecalis*	CIP 103015	CIP	BHI	37	No
*Leuconostoc mesenteroides*	ATCC 14935	ATCC ^3^	MRS	30	Yes
*Listeria innocua*	CIP 80.11	CIP	BHI	37	No
*Listeria ivanovii*	CIP 78.42T	CIP	BHI	37	No
*Listeria monocytogenes*	DSMZ 19094	DSMZ	BHI	37	No
*Listeria monocytogenes*	ATCC 35152	ATCC	BHI	37	No
*Mucor circinelloides*	UBOCC-A-112187	UBOCC ^4^	MEA ^7^	25	No
*Mucor racemosus*	UBOCC-A-111130	UBOCC	MEA	25	No
*Penicillium solitum*	UBOCC-A-111055	UBOCC	MEA	25	No
*Pseudomonas aeruginosa*	ATCC 27853	ATCC	BHI	30	No
*Pseudomonas fluorescens*	CIP 69.13	CIP	BHI	30	No
*Staphylococcus aureus*	DSMZ 13661	DSMZ	BHI	37	No
*Staphylococcus aureus*	CIP 76.25	CIP	BHI	37	No
*Salmonella* ser. Enteritidis	CIP 82.97	CIP	BHI	37	No
*Salmonella* ser. Typhimurium	CIP 104115	CIP	BHI	37	No
*Yarrowia lipolytica*	UBOCC-A-211004	UBOCC	YPD ^8^	25	No
*Yersinia enterocolitica* subsp. *enterocolitica*	DSMZ 4780	DSMZ	BHI	26	No

^1^ Collections of Institut Pasteur (CIP), ^2^ Leibniz Institute Deutsche Sammlung von Mikroorganismen und Zellkulturen, ^3^ American Type Culture Collection, ^4^ Cultures de l’Université de Bretagne Occidentale, ^5^ brain heart infusion, ^6^ de Man Rogosa and Sharpe, ^7^ malt extract agar, ^8^ yeast potato dextrose.

**Table 4:** Amplicon sequence variants (ASVs) matrix count (%) determined with *purR* gene analysis.

	Abundance (%)
ASV_1	99.86
ASV_2	0.13
ASV_3	0.005
ASV_4	0.005
ASV_5	0.002

**Table 5:** Growth conditions and number of collected isolates used to build the bacterial library.

Growth Conditions			Number of Collected Isolates
Aerobic atmosphere	28 °C	BHI agar	128
		MRS agar	192
		M17 agar	192
		MRS agar + 2% NaCl	96
		MRS agar + 4% NaCl	96
		MRS agar + 6.5% NaCl	96
		BHI agar + 6.5% NaCl	96
Microaerophilic atmosphere	28 °C	BHI agar	48
		MRS agar	192
		M17 agar	192
Aerobic atmosphere	22 °C	BHI agar	96
	37 °C	MRS agar	96
	44 °C	MRS agar	96
**Total**			**1616**

**Table 6:** Phenotypic characteristics of UMSC *Lactococcus* isolates. Phenotypic characteristics of two strain types, ATCC 19,435 and ATCC 19,257; data were extracted from Buron-Moles et al. and Yu et al. [14,53]. Phenotypic data of the type strain IL1403 was extracted from Siezen et al. [54].

Carbohydrate	A Group (*n* = 13) ^a^	B Group (*n* = 2) ^b^	C Group (*n* = 2) ^c^	D group (*n* = 1) ^d^	ATCC 19435 ^e^	ATCC 19257 ^f^	IL1403 ^h^
Strain origin	Raw milk	Raw milk	Raw milk	Raw milk	Milk	Cream	Dairy
Control	-	-	-	-	-	-	-
D-ribose	+	+	+	+	+	-	+
L-arabinose	-	-	-	-	-	-	-
D-xylose	+	-	+	+	+	-	-
D-galactose	+	+	+	+	+	-	+
D-glucose	+	+	+	+	+	+	+
D-fructose	+	+	+	+	+	+	+
D-mannose	+	+	+	+	w ^g^	+	+
D-mannitol	-	+	-	+	-	-	-
D-sorbitol	-	+	-	-	-	-	-
Arbutin	+	+	+	+	+	-	+
Salicin	+	+	+	+	+	-	+
D-cellobiose	+	+	+	+	+	-	+
D-maltose	+	+	+	+	+	-	+
D-lactose	+	+	+	+	+	+	w
D-melibiose	-	-	-	-	-	-	-
D-sucrose	-	+	+	+	-	-	-
D-trehalose	+	+	+	+	+	-	+
D-raffinose	-	-	-	-	-	-	-
Starch	-	-	-	-	-	-	-
D-tagatose	-	+	-	-	-	-	-
Percentage of carbohydrates (*n* = 49) utilized per strain (%)	33	45	35	35	29	10	29

L_x_: *Lactococcus lactis* species, ^a^ A Group: L1 to L6, L10 to L13 and L15 to L17, ^b^ B Group: L7 and L8, ^c^ C Group: L14 and L18, ^d^ D Group: L9, ^e^ *L*. *lactis* subsp. *lactis* ATCC 19,435 [14,52], ^f^ *L. lactis* subsp. *cremoris* ATCC 19,257 [14,52], ^g^ weakly positive, ^h^ *L. lactis* subsp. *lactis* IL1403 [54].

**Table 7:** Antimicrobial spectrum of CFS and NCFS produced by LAB isolates against related LAB, food-spoilage and foodborne pathogens by the well diffusion assay.

Tested Isolates	*S.* ser. Typhimurium CIP 104115		*S. aureus* DSMZ 13661		*C. maltaromaticum* CIP 103158		*S.* ser. Enteritidis CIP 82.97		*S. aureus* CIP 76.25		*E. faecalis* CIP 103015		*L. innocua* CIP 80.11	
	CFS	NCFS	CFS	NCFS	CFS	NCFS	CFS	NCFS	CFS	NCFS	CFS	NCFS	CFS	NCFS
1	+	-	-	-	-	-	-	-	+	-	-	-	+	-
3	+	-	-	-	-	-	-	-	-	-	-	-	+	-
4	-	-	+	-	-	-	-	-	-	-	+	-	+	-
5	+	-	-	-	-	-	-	-	-	-	+	-	+	-
6	+	-	-	-	-	-	-	-	-	-	+	-	+	-
8	-	-	+	-	-	-	-	-	+	-	+	-	+	-
9	-	-	+	-	-	-	-	-	-	-	-	-	+	-
10	-	-	+	-	-	-	-	-	-	-	-	-	+	-
11	-	-	+	-	-	-	-	-	-	-	+	-	+	-
12	-	-	+	-	+	-	-	-	-	-	-	-	+	-
13	-	-	+	-	-	-	-	-	-	-	+	-	+	-
14	+	-	+	-	-	-	-	-	+	-	+	-	+	+
15	+	-	+	-	-	-	-	-	+	-	+	-	+	+
16	-	-	+	-	-	-	-	-	-	-	+	-	+	-
17	-	-	+	-	-	-	-	-	+	-	-	-	+	-
18	+	-	+	-	-	-	-	-	-	-	+	-	+	-
20	+	-	-	-	-	-	-	-	-	-	+	-	+	-
23	+	-	-	-	-	-	-	-	+	-	-	-	+	-
24	-	-	-	-	-	-	+	-	+	-	-	-	+	-
25	+	-	-	-	-	-	-	-	+	-	-	-	+	-
26	+	-	-	-	-	-	-	-	+	-	-	-	+	-
27	+	-	-	-	-	-	-	-	+	-	-	-	+	-
30	-	-	+	-	-	-	-	-	+	-	-	-	+	-
32	-	-	-	-	-	-	+	-	+	-	-	-	+	-
33	-	-	+	-	-	-	-	-	+	-	-	-	+	-
34	+	-	-	-	-	-	-	-	+	-	-	-	+	-

CFS: cell-free supernatant, NCFS: neutralized cell-free supernatant (adjusted to pH 7), +: presence of an inhibition halo (>5 mm), -: no inhibition halo.

**Table 8:** Antimicrobial activities of CFSs collected from the culture of *L. lactis* subsp. *lactis*-isolate 14 cultivated with and without *S.* ser. Typhimurium CIP 104,115 or *S. aureus* DSMZ 13661. Plates were examined for clear inhibition halo (mm), evaluated by the CFS agar well diffusion assay. Largest inhibition halos are recorded below. CFSs were collected after 16 h of growth for *S. ser.* Typhimurium CIP 104,115 assays and after 32 h of growth for *S. aureus* DSMZ 13,661 assays.

Tested CFS	*S.* ser. Typhimurium CIP 104115	*Lactobacillus Sakei* CIP 104494	*S. aureus* DSMZ 13661	*L. innocua* CIP 80.11	*L. lactis* IL1403 ^d^
*L. lactis* isolate 14	8.0 ± 0.7 ^a^	12.0 ± 0.7	8.0 ± 0.0	24.0 ± 0.7	13.0 ± 1.4
*S.* ser. Typhimurium CIP 104115	/^b^	/	/	/	/
Isolate 14 + *S.* ser. Typhimurium CIP 104115	11.0 ± 0.0	14.0 ± 1.4	ND ^c^	22.0 ± 1.4	15.0 ± 2.12
*S. aureus* DSMZ 13661	/	10.0 ± 2.12	/	/	/
Isolate 14 + *S. aureus* DSMZ 13661	ND	11.0 ± 1.4	14.0 ± 0.0	18.0 ± 0.7	17.0 ± 0.7

^a^ Mean ± SD from duplicate determinations, ^b^: no inhibition halo, ^c^ ND: not determined, ^d^ plasmid-free strain; indicator strain for lactococcin B-like bacteriocin [57].

## Supplementary Materials

The following are available online at https://www.mdpi.com/2304-8158/9/5/622/s1, Figure S1. Carbohydrate metabolism tested using the API 50 CH system (BioMérieux, Marcy l’Etoile, France). A blue dot highlights a positive response to carbohydrate degradation. Figure S2. Pairwise average nucleotide identity (ANI) values across six *Lactococcus* genomes and the reference genome *L. lactis* subsp. *lactis* IL1403. Colors in the heatmap represent pairwise ANI values, with a gradient from blue (low identity) to green (high identity). Table S1. Antimicrobial spectrum of LAB candidate isolates against related LAB, food-spoilage and foodborne pathogens evaluated by the agar well diffusion assay. Plates were recorded for inhibition halos (mm).
