# Supplementary material for: Lactococcus lactis Diversity Revealed by Targeted Amplicon Sequencing of purR Gene, Metabolic Comparisons and Antimicrobial Properties in an Undefined Mixed Starter Culture Used for Soft-Cheese Manufacture
Source: Foods. 2020 May 13;9(5):622. doi: 10.3390/foods9050622 (PMC7278722; doi:10.3390/foods9050622)
Supplement: Supplementary file 1 [file foods-09-00622-s001.zip › Figure S1_Article_Sabrina Saltaji.pdf]

Supplementary data

	L1	R1	S1	L2	L3	L4	L5	L6	E1	E2	L7	L8	E3	S2	L9	L10	L11	L12	L13	L14	L15	L16	L17	L18	E4	E5
T																										
GLY											●	●	●												●	●
ERY																										
DARA																										
LARA																										
RIB	●	●		●	●	●	●	●	●	●	●	●	●	●	●	●	●	●	●	●	●	●	●	●	●	●
DXYL	●	●		●	●	●	●	●	●	●			●	●	●	●	●	●	●	●	●	●	●	●		
LXYL																										
ADO																										
MDX																										
GAL	●	●		●	●	●	●	●	●	●	●	●	●	●	●	●	●	●	●	●	●	●	●	●	●	●
GLU	●	●	●	●	●	●	●	●	●	●	●	●	●	●	●	●	●	●	●	●	●	●	●	●	●	●
FRU	●	●	●	●	●	●	●	●	●	●	●	●	●	●	●	●	●	●	●	●	●	●	●	●	●	●
MNE	●	●		●	●	●	●	●	●	●	●	●	●	●	●	●	●	●	●	●	●	●	●	●	●	●
SBE																										
RHA																										
DUL																										
INO																										
MAN		●	●								●	●	●	●	●										●	●
SOR											●	●	●												●	●
MDM																										
MDG																										
NAG	●	●		●	●	●	●	●	●	●	●	●	●	●	●	●	●	●	●	●	●	●	●	●	●	●
AMY	●	●		●	●	●	●	●	●	●	●	●	●	●	●	●	●	●	●	●	●	●	●	●	●	●
ARB	●	●		●	●	●	●	●	●	●	●	●	●	●	●	●	●	●	●	●	●	●	●	●	●	●
ESC	●	●		●	●	●	●	●	●	●	●	●	●	●	●	●	●	●	●	●	●	●	●	●	●	●
SAL	●	●		●	●	●	●	●	●	●	●	●	●	●	●	●	●	●	●	●	●	●	●	●	●	●
CEL	●	●		●	●	●	●	●	●	●	●	●	●	●	●	●	●	●	●	●	●	●	●	●	●	●
MAL	●	●		●	●	●	●	●	●	●	●	●	●	●	●	●	●	●	●	●	●	●	●	●	●	●
LAC	●	●		●	●	●	●	●	●	●	●	●	●	●	●	●	●	●	●	●	●	●	●	●	●	●
MEL		●																								
SAC		●	●								●	●	●	●	●					●				●	●	●
TRE	●	●	●	●	●	●	●	●	●	●	●	●	●	●	●	●	●	●	●	●	●	●	●	●	●	●
INU																										
MLZ											●	●	●												●	●
RAF		●																								
AMD		●																								
XLT																										
GEN	●	●		●	●	●	●	●	●	●	●	●	●	●		●	●	●	●	●	●	●	●	●	●	●
TUR																										
LYX																										
TAG											●	●	●												●	●
DFUC																										
LFUC																										
DARL																										
LARL																										
GNT											●	●	●												●	●
2KG																										
5KG																										

Figure S1
Carbohydrate metabolism tested using the API 50 CH system (BioMérieux, Marcy l'Etoile, France). A blue dot highlights a positive response to carbohydrate degradation.

T : control, GLY, glycerol, ERY : erythritol, DARA : D-arabinose, LARA : L-arabinose, RIB : D-ribose, DXYL : D-xylose, LXYL : L-xylose, ADO : D-adonitol, MDX : methyl-βD-xylopyranoside, GAL : D-galactose, GLU : D-glucose, FRU : D-fructose, MNE : D-mannose, SBE : L-sorbose, RHA : L-rhamnose, DUL : dulcitol, INO : inositol, MAN : D-mannitol, SOR : D-sorbitol, MDM : methyl-αD-Mannopyranoside, MDG : methyl-αD-Glucopyranoside, NAG : N-AcetylGlucosamine, AMY : amygdalin, ARB : arbutin, ESC : esculin, SAL : salicin, CEL : D-cellobiose, MAL : D-maltose, LAC : D-lactose, MEL : D-melibiose, SAC : D-sucrose, TRE : D-trehalose, INU : D-inuline, MLZ : D-melezitose, RAF : D-raffinose, AMD : amidon, GLYG : glycogen, XLT : xylitol, GEN : gentiobiose, TUR : D-turanose, LYX : D-Lyxose, TAG : D-tagatose, DFUC : D-fucose, LFUC : L-fucose, DARL : D-arabitol, LARL : L-arabitol, GNT : potassium gluconate, 2KG : potassium 2-ketogluconate, 5KG : potassium 5-ketogluconate.
L: Lactococcus lactis, R: *Lactococcus raffinolactis*, E: *Enterococcus faecalis*, S: *Staphylococcus warneri* (S)
