# Supplementary material for: Lactococcus lactis Diversity Revealed by Targeted Amplicon Sequencing of purR Gene, Metabolic Comparisons and Antimicrobial Properties in an Undefined Mixed Starter Culture Used for Soft-Cheese Manufacture
Source: Foods. 2020 May 13;9(5):622. doi: 10.3390/foods9050622 (PMC7278722; doi:10.3390/foods9050622)
Supplement: Supplementary file 1 [file foods-09-00622-s001.zip › Figure S2_ANI_Sabrina Saltaji.pdf]

Supplementary data

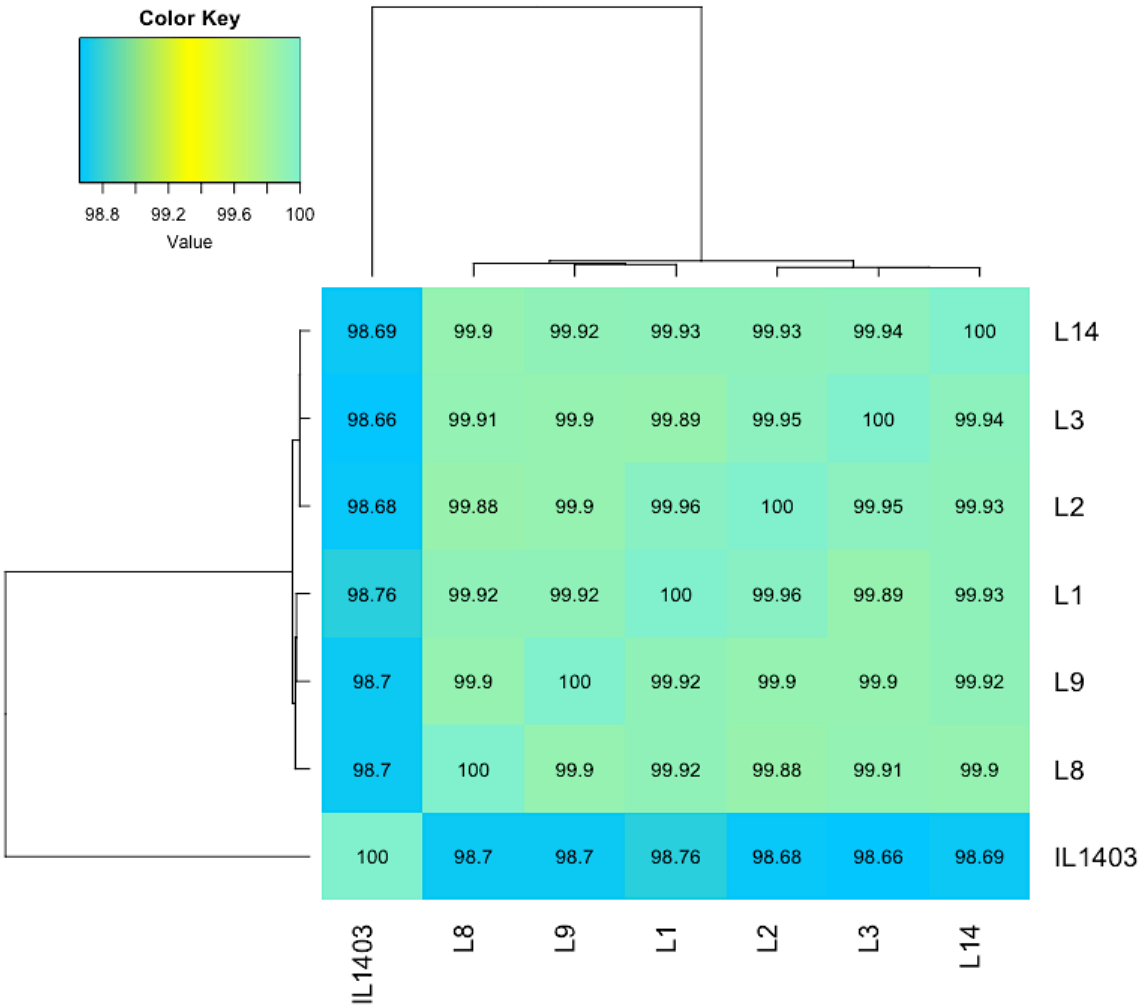

Figure S2
Pairwise average nucleotide identity (ANI) values across six *Lactococcus* genomes and the reference genome *L. lactis* subsp. *lactis* IL1403. Colors in the heatmap represent pairwise ANI values, with a gradient from blue (low identity) to green (high identity).
