# Supplementary material for: Lactococcus lactis Diversity Revealed by Targeted Amplicon Sequencing of purR Gene, Metabolic Comparisons and Antimicrobial Properties in an Undefined Mixed Starter Culture Used for Soft-Cheese Manufacture
Source: Foods. 2020 May 13;9(5):622. doi: 10.3390/foods9050622 (PMC7278722; doi:10.3390/foods9050622)
Supplement: Supplementary file 1 [file foods-09-00622-s001.zip › Table S1_Antimicrobial activities_Saltaji.pdf]

Supplementary data

Table S1
Antimicrobial spectrum of LAB candidate isolates against related LAB, food-spoilage and foodborne pathogens evaluated by the agar well diffusion assay. Plates were recorded for inhibition halos (mm).

Tested isolates	*S.* ser. Typhimurium CIP 104115	*S. aureus* DSMZ 13661	*C.* maltaromaticum CIP 103158	*S.* ser. Enteritidis CIP 82.97	*S. aureus* CIP 76.25	*E. faecalis* CIP 103015	*L. innocua* CIP 80.11
1	6.5 ± 0.7[c]	/[b]	/	/	6.5 ± 0.7	/	6.5 ± 0.7
2	/	/	/	/	7.5 ± 0.7	/	6.5 ± 0.7
3	6.0 ± 0.0	/	/	/	/	/	7.5 ± 0.7
4	/	13.5 ± 0.7	/	/	/	5.5 ± 0.7	7.0 ± 1.4
5	6.5 ± 0.7	/	/	/	/	5.5 ± 0.7	7.0 ± 1.4
6	6.5 ± 0.7	/	/	/	/	6.0 ± 1.4	7.0 ± 0.0
7	/	/	/	/	/	8.5 ± 0.7	8.5 ± 0.7
8	/	8.5 ± 0.7	/	/	7.5 ± 0.7	11.5 ± 2.1	8.5 ± 0.7
9	/	6.5 ± 0.7	/	/	/	/	7.5 ± 0.7
10	/	8.5 ± 0.7	/	/	/	/	8.5 ± 0.7
11	/	17.0 ± 1.4	/	/	/	11.5 ± 0.7	14.5 ± 0.7
12	/	13.5 ± 0.7	19.0 ± 1.4	/	/	/	12.5 ± 0.7
13	/	19.5 ± 0.7	/	/	/	8.5 ± 0.7	16.5 ± 0.7
14	7.5 ± 0.7	15.5 ± 2.1	/	/	13.5 ± 0.7	7.5 ± 0.7	18.0 ± 0.0
15	6.0 ± 0.0	18.0 ± 2.8	/	/	12.0 ± 0.0	6.5 ± 0.7	14.5 ± 0.7
16	/	16.5 ± 2.1	/	/	/	13.5 ± 0.7	11.0 ± 1.4
17	/	13.0 ± 1.4	/	/	11.5 ± 0.7	/	12.5 ± 2.1
18	7.0 ± 1.4	13.5 ± 0.7	/	/	/	11.0 ± 1.4	13.5 ± 0.7
19	/	/	/	/	/	11.0 ± 1.4	11.5 ± 0.7
20	6.5 ± 0.7	/	/	/	/	15.5 ± 2.1	9.5 ± 0.7
21	/	/	/	/	/	12.5 ± 3.5	9.5 ± 0.7
22	/	/	/	/	14.5 ± 0.7	/	13.0 ± 1.4
23	7.5 ± 0.7	/	/	/	15.0 ± 1.4	/	13.0 ± 1.4
24	/	/	/	7.5 ± 0.7	7.5 ± 0.7	/	15.5 ± 0.7
25	7.0 ± 1.4	/	/	/	6.5 ± 0.7	/	10.0 ± 1.4
26	6.5 ± 0.7	/	/	/	7.0 ± 1.4	/	11.0 ± 4.2
27	7.5 ± 0.7	/	/	/	11.0 ± 2.8	/	15.5 ± 0.7
28	/	/	/	/	12.5 ± 0.7	/	16.0 ± 2.8
29	/	/	/	/	9.0 ± 0.0	/	13.0 ± 1.4
30	/	11.5 ± 0.7	/	/	16.0 ± 2.8	/	16.5 ± 0.7
31	/	/	/	/	12.0 ± 2.8	/	9.0 ± 0.0
32	/	/	/	/	8.5 ± 0.7	/	7.5 ± 0.7
33	/	5.5 ± 0.7	/	/	13.0 ± 1.4	/	15.5 ± 0.7
34	7.5 ± 0.7	/	/	/	11.0 ± 1.4	/	16.0 ± 1.4
(+)	6.0 ± 1.4	7.5 ± 3.5	6.5 ± 2.12	5.5 ± 0.0	16.5 ± 2.1	13.0 ± 1.4	15.5 ± 2.1

[a] Mean ± SD from duplicate determinations
[b] / : no inhibition halo
(+): positive control *L. sakei* subsp. *sakei* CIP 104494
